# Safety of the Entomopathogenic Fungus *Beauveria bassiana* for Wild and Laboratory-Reared *Chrysoperla lucasina* Strains

**DOI:** 10.3390/insects15080576

**Published:** 2024-07-29

**Authors:** Walaa Morda, Maria Tiziana Nuvoli, Luca Ruiu

**Affiliations:** Department of Agricultural Sciences, University of Sassari, 07100 Sassari, Italy; w.morda@studenti.uniss.it (W.M.); mtnuvoli@uniss.it (M.T.N.)

**Keywords:** biological control, predator, pest management, microbials, compatibility, IPM

## Abstract

**Simple Summary:**

Natural predators such as the chrysopid *Chrysoperla lucasina* occur in agroecosystems, where they naturally contribute to containing pest populations. Their biological properties can be leveraged by mass rearing in the laboratory and releasing them in the field, where they can express their predatory potential against pest species. To ensure the preservation of these beneficial species, plant protection products should be harmless to them. Accordingly, the use of biopesticides based on microbial entomopathogens, such as the fungus *Beauveria bassiana* strain ATCC 74040, is promoted as they are generally regarded as safe to non-target species. However, such safety should be demonstrated case by case. In addition, safety should be ensured for both the wild species and those released for biological control. This study involved experiments including laboratory-reared and wild *C. lucasina* larvae and determined only slight or no effects of *B. bassiana* on their survival, immature development, adult emergence, and reproductive potential. These findings highlight the compatibility of the predator and this strain of *B. bassiana*, emphasising the opportunity for their combined use in environmentally friendly pest management strategies.

**Abstract:**

The need to reduce the impact of plant protection products on agroecosystems fosters the use of augmentative biological control involving the release of beneficial species into the field, the employment of entomopathogenic microbials, and the protection of naturally occurring biocontrol agents. This study aimed to investigate the compatibility of the entomopathogenic fungus *Beauveria bassiana* with the generalist insect predator *Chrysoperla lucasina*, in comparative experiments involving a laboratory-reared and a wild chrysopid strain. The larvae of the predators were exposed to different concentrations of fungal conidia up to a concentration of 10^7^ conidia/mL by contact and ingestion. The treated insects showed only slight differences in terms of survival and immature development time compared to the untreated control insects. A significant decrease in the proportion of the male adults of *C. lucasina* that emerged from the laboratory-reared larvae that were exposed to higher concentrations of the fungus suggested a potentially different susceptibility between the sexes. A slightly lower adult emergence rate was observed in the wild strain, while no significant differences were recorded in the adult reproductive performance. These findings indicate that the *B. bassiana* strain ATCC 74040, at concentrations commonly used in the field, did not pose a significant risk to *C. lucasina* and can be safely used in combination with this predator for sustainable pest management.

## 1. Introduction

The need to protect agricultural production systems from the deleterious action of pests while minimizing the impact on the environment fosters the use of bio-based solutions, among which biological control agents (BCAs) like predators and parasitoids are often employed successfully. This can be achieved with different techniques, including the release of beneficial insects or the enhancement of naturally occurring antagonists through ecosystem manipulation [1]. Accordingly, several chrysopid species occur on a variety of crops, where they act as generalist predators regulating the action of various pests, including aphids, thrips, and whiteflies. *Chrysoperla lucasina* (Lacroix) (Neuroptera: Chrysopidae), is a member of the *Chrysoperla carnea* (Stephens) group, commercialized as a BCA for inundative biocontrol programs [2]. This insect is therefore present both in the wild form and as a product of artificial breeding. Consequently, ecosystem management interventions should be compatible with this useful entomofauna [3].

Another bio-based approach for pest management is the use of microbials like entomopathogenic bacteria, viruses, fungi, and nematodes (EPNs), which cause diseases that specifically target pests. Among these, the fungus *Beauveria bassiana* (Bals.-Criv.) Vuill. is widely used for its efficacy and broad spectrum of action against a variety of crop pests across different orders, including Lepidoptera, Coleoptera, Heteroptera, Homoptera, Diptera, and Hymenoptera [4,5]. The action of *B. bassiana* typically relies on the adhesion of aerial conidia to the hydrophobic surface of a host’s cuticle, followed by germination and penetration through the cuticle to reach the rich haemocoel environment where the fungus can produce further propagules and hyphae that infect diverse host organs and tissues, and release metabolites to intoxicate the host and overcome its immune system, thus leading to the insect’s death. If environmental conditions are favourable, the fungus can produce conidiophore-carrying aerial conidia on the surface of the dead host, which will ensure its dissemination [6,7]. Several studies have demonstrated the successful use of *B. bassiana* in various agroecosystems with little ecological impact. The compatibility of this fungus with most of its natural enemies has been reported [8], including Coleoptera, Hemiptera, Collembola [9], and Hymenoptera [10,11]. On the other hand, this fungus was reported to interfere with the recognition mechanisms of honeybee nestmates [12]. This highlights the importance of conducting extensive studies to assess the potential impact on beneficial species not only in terms of acute toxicity, but also in terms of the behavioural or developmental effects that could occur even with sublethal exposure.

The purpose of this study was to investigate the possible impact of *B. bassiana* on wild and laboratory-reared populations of the predatory lacewing *C. lucasina* when exposed to different concentrations of the entomopathogenic fungus *B. bassiana*. Developmental and life-table parameters in treated and untreated insects were also compared.

## 2. Materials and Methods

### 2.1. Fungal Preparations

The entomopathogenic fungus *Beauveria bassiana* strain ATCC 74040, commercially available as the active substance of the product Naturalis^®^ (CBC Europe S.r.l., Nova Milanese, Italy), was used in this study [13]. Microbial cultures were routinely grown at 25 ± 1 °C on potato dextrose agar (PDA) (Liofilchem, Teramo, Italy) to ensure continuous availability during the experiments [13]. The aerial conidia that were used in bioassays were scraped from fresh PDA plates into a 0.02% Tween 80 solution. The resulting conidia suspensions were checked under a phase microscope (Zeiss, Novara, Italy) for purity and quantified in a Neubauer chamber (Blaubrand, Wertheim, Germany). Distilled water was used to adjust the concentration of the suspension as needed for bioassays. The conidia suspensions were used in bioassays immediately after their preparation to ensure the highest viability (>90%).

### 2.2. Insect Rearing

During this study, two colonies of *C. lucasina* were maintained in the laboratory, of which the first was normally used in augmentative biocontrol programs (laboratory strain) and the second (wild strain) was initiated using wild-caught specimens from the countryside of Olmedo (Sardinia, Italy). The insects were reared at 25 °C with a photoperiod of L16:D8 in compliance with previously described methods [14] with necessary modifications [15].

Larvae (mealworms) of *Tenebrio molitor* L. (Coleoptera: Tenebrionidae), used in bioassays or as lacewing prey, were provided by the insect rearing facility of the Department of Agricultural Sciences of the University of Sassari (Italy) [16].

### 2.3. Insect Bioassays

#### 2.3.1. Survival Bioassays

**Figure 1:**
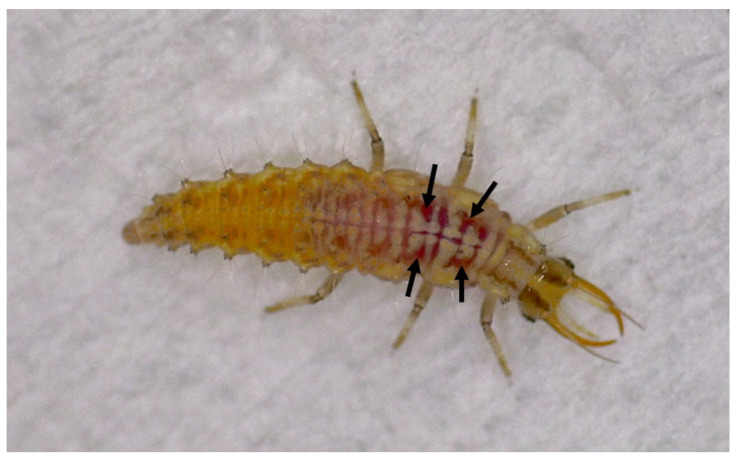
*Chrysoperla lucasina* larva fed with 20% fructose drops containing a food dye with evidence of red coloration showing through the gut (arrows).

The potential lethal effects of *B. bassiana* were evaluated by exposing chrysopid larvae to fructose drops containing fungal conidia, so as to encourage their contact and ingestion. To set up a reliable experimental model, preliminary observations were conducted on larvae that were exposed for 24 h to 20% fructose drops (4 µL) with a red vegetable-based food dye [17]. During this period, the larvae were regularly observed making contact with the droplets and feeding on them. Thereafter, the larvae were observed under a phase microscope to detect the presence of the dye in their gut, which confirmed the ingestion of the coloured liquid and thus also the fungal conidia when present (Figure 1).

According to this method, first instar larvae of *C. lucasina* were individually placed in transparent plastic pots (2 cm diameter × 3 cm high) with a drop (4 µL) of 20% fructose suspension containing a specific conidia concentration (10^7^, 10^6^, or 10^5^/mL) or without conidia (control). Treated and control larvae were incubated at 25 °C and maintained in the following period on a diet consisting of two mealworms per individual, provided every other day. Each treatment involved four replicates (10 individuals each), and the insects were inspected daily, assessing mortality after 7 days. Individual maintenance of larvae was necessary to avoid cannibalism.

To assess the virulence of the conidia suspension stocks that were used in the experiments with chrysopids, additional bioassays were conducted in parallel on *T. molitor* larvae. For this purpose, groups of 10 mealworms were immersed for 30 s in *B. bassiana* suspensions at different concentrations (10^7^, 10^6^, or 10^5^ conidia/mL) and incubated at 25° C on sterile plates with filter paper for 96 h, finally assessing larval mortality. The experimental design involved 4 replicates for each treatment and for the untreated control group (larvae immersed in sterile water).

All of the experiments were repeated over time with at least three different cohorts of insects and *B. bassiana* batches from different laboratory preparations.

#### 2.3.2. Bioassays on Life-Table Parameters

According to the experimental design previously described, individually reared chrysopid larvae that were exposed to different concentrations of fungal conidia (10^7^, 10^6^, or 10^5^ conidia/mL) or left untreated (control), were maintained individually in pots that were inspected daily and provided with two mealworms per individual on alternate days, to follow their preimaginal development until pupation and adult emergence. After recording insect survival and the developmental stage duration, the emerging adults were transferred into new cages (10 cm diameter and 10 cm high) at a 1:1 sex ratio, allowed to mate, and were fed *ad libitum* with pollen and water. Each cage included a removable paper sheet in the top side where eggs were regularly oviposited by females [15]. The paper sheet was replaced daily to allow continuous egg counts over the following three-week period. The insect mortality in each cage was also recorded [16].

Four groups (replicates) of 10 eggs from each cage (treated and control) were periodically analysed to assess their viability. For this purpose, eggs were placed individually in a separate pot and monitored over a week until hatching. These analyses were performed 4 times during the oviposition period.

### 2.4. Data Analysis and Statistics

Data were analysed using R software, version 4.1.2 [18]. Two-way ANOVA (factors: treatment and *C. lucasina* strain) was used to analyse the *C. lucasina* data on survival, larval and pupal development time, adult emergence, sex ratio, oviposition, and egg hatching. The percentage data were arcsin-squareroot-transformed before analysis [19]. The assumption of homogeneity of variance across the groups was confirmed by Levene’s test. One-way ANOVA was used to analyse the mortality data for *Tenebrio molitor* larvae. A post-hoc comparison based on the Tukey test [20] was used when significant main effects were detected.

## 3. Results

### 3.1. Survival Bioassays

#### 3.1.1. Bioassays with *Chrysoperla lucasina*

**Figure 2:**
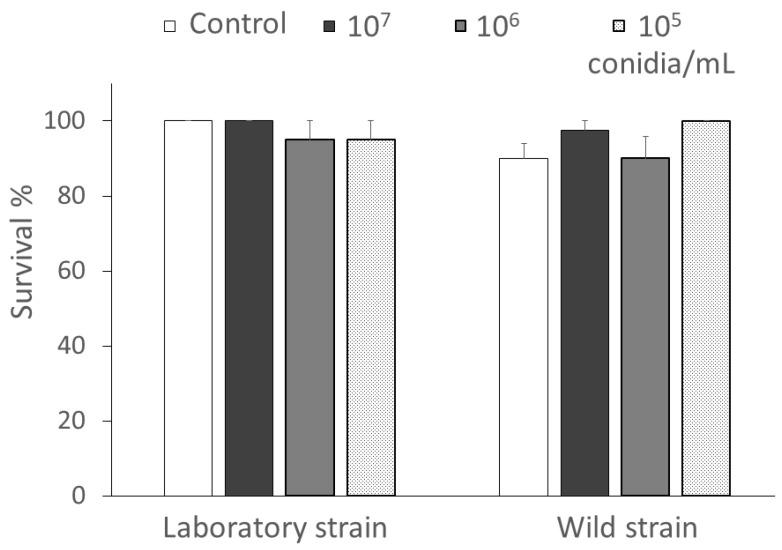
Survival percentage (mean ± SE) of *Chrysoperla lucasina* larvae exposed to different concentrations (10^7^, 10^6^, or 10^5^ conidia/mL) of *Beauveria bassiana* conidia. Means were not significantly different (two-way ANOVA on transformed data, *p* > 0.05).

The survival of newly hatched *C. lucasina* larvae that were exposed by contact and fed on fructose drops containing different concentrations of *B. bassiana* conidia appeared to be consistently above 90% after 7 days and without significant differences compared with the untreated control group (F_3,24_ = 0.99; *p* = 0.4100). Furthermore, no differences were detected in the comparison between laboratory and wild strains (F_1,24_ = 1.94; *p* = 0.1750), nor in the interaction between treatments and chrysopid strains (F_3,24_ = 1.71; *p* = 0.1920) (Figure 2).

#### 3.1.2. Bioassays with *Tenebrio molitor*

**Figure 3:**
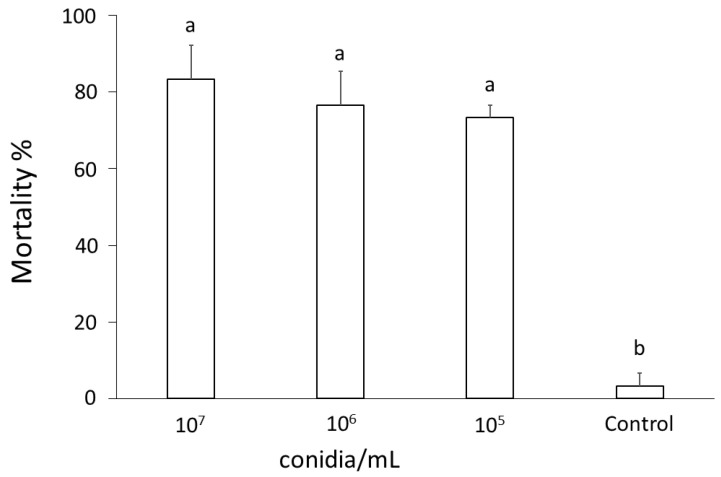
Mortality percentage (mean ± SE) of *Tenebrio molitor* larvae exposed to different concentrations (10^7^, 10^6^, or 10^5^ conidia/mL) of *Beauveria bassiana* conidia. Different letters above bars indicate significantly different means (one-way ANOVA on transformed data, *p* < 0.001).

In virulence bioassays with *T. molitor*, aimed at assessing the entomopathogenic potential of *B. bassiana* against insects, conidia that were brought into contact with the mealworms’ integument caused an average mortality exceeding 80% at 96 h after treatment at all concentrations assayed (10^7^, 10^6^, and 10^5^ conidia/mL), while low mortality (<4%) was observed in the untreated control group (F_3,11_ = 18.64; *p* < 0.001) (Figure 3).

### 3.2. Bioassays on Life-Table Parameters

The average larval and pupal development time and adult emergence rate of *C. lucasina* individuals that were exposed to different concentrations of *B. bassiana* conidia at the first larval instar are reported in Table 1. The development time was not significantly different for the treated compared to the control larvae (F_3,24_ = 0.53; *p* = 0.6652), and for the wild compared to the laboratory strain (F_3,24_ = 3.47; *p* = 0.0746), and no differences between treatments (F_3,24_ = 0.01; *p* = 0.9988) and strains (F_3,24_ = 0.16; *p* = 0.9203) were observed in the duration of the pupal stage.

A slightly lower, though not significant, adult emergence rate was observed in the treated compared with the control groups of both the laboratory and wild strains of *C. lucasina* (F_3,24_ = 0.40; *p* = 0.7514). Adult emergence was significantly higher in the lab-reared in comparison with the wild chrysopids (F_3,24_ = 5.94; *p* = 0.0217).

The sex ratio was similar in the untreated laboratory-reared and untreated wild *C. lucasina*. The laboratory strain had an adult sex ratio of 73% male in the highest inoculum treatment that differed significantly from that of the control group or other inoculum concentrations (F_3,24_ = 5.35; *p* = 0.0269). Other variations in differently treated groups were not significant (F_3,24_ = 1.38; *p* = 0.2860).

The average number of eggs per female that were laid by individuals that emerged from the larvae that were exposed to *B. bassiana* conidia at different concentrations was not affected by treatment (F_3,24_ = 0.23; *p* = 0.8694) or the *C. lucasina* strain (F_3,24_ = 3.96; *p* = 0.0580). Similarly, no differences were observed in the egg-hatching rate between treatments (F_3,24_ = 0.26; *p* = 0.8510) or strains (F_3,24_ = 1.60; *p* = 0.2160) (Table 2).

## 4. Discussion

The need to limit the use of broad-spectrum chemical insecticides for pest management in agroecosystems is well recognized for the preservation of entomophagous species. The mass breeding of beneficial species for field release is also required for augmentative biological control [21].

However, the quality of laboratory-reared insects is frequently lower, in terms of their biological characteristics, biotic potential, and predator or parasitoid performance, due to their reduced genetic diversity and the artificial rearing conditions [22]. Though this aspect is the subject of continuous studies aimed at improving the quality of these insects, it follows that a significant difference in their ability to adapt to different environmental conditions may exist when comparing between laboratory-reared and wild strains [23].

Another aspect that influences the quality of the biocontrol activity that is carried out in the field by the beneficial species, and which could highlight differences between lab-reared and wild strains, is their susceptibility to biotic factors, including the entomopathogenic agents that are used as biopesticides [4,24].

Among the latter is the fungus *Beauveria bassiana*, whose entomopathogenic action against insects in different orders begins with the adhesion of aerial conidia to the host’s integument, followed by mechanical penetration through the cuticle by means of special structures that are aided by specific enzymes (chitinases and proteases), which allows the fungus to reach the insect’s haemocoel where it finds nutrients that promote its development and further spread in the insect’s body. The pathogenic process ends with the production of conidiophore branches carrying new aerial conidia that will promote spreading in the environment [25]. The action of the fungus is therefore nonspecific and requires favourable environmental conditions for its accomplishment. It therefore becomes essential to verify its selectivity and compatibility towards beneficial insects [4].

The hypothesis in the present study was that larvae of the predator *C. lucasina,* that normally move about the plant in search of prey, come into contact with droplets of the plant protection product containing *B. bassiana* conidia. Under our experimental conditions, it has been shown that these larvae, in addition to coming into contact with the fungus, can also ingest it, adding to the above-described action by contact also an action by ingestion [26].

However, at the concentrations assayed (equal to and less than 10^7^ conidia/mL), which reflect those that the predator might encounter in the field in accordance with the application doses indicated in the biopesticide product label, no effect on the survival of either the laboratory or wild strains was observed. This result aligns with several studies in which different chrysopid species were exposed to other strains of *B. bassiana* [27,28].

Greater variability was observed in the sex ratio of adults that emerged from early age larvae that were exposed to the fungus. On this, there were no substantial differences between the two strains of *C. lucasina*, although a significant reduction in the proportion of female individuals was observed as an effect of treatment at the higher concentration, which supports sex-specific differences in their susceptibility to infection.

Alongside a wide individual variability, the fecundity of *C. lucasina* females that emerged from the treated larvae was not affected by the treatments and no differences between the lab and wild strains emerged, which corroborates several other studies with chrysopids reporting slight to non-significant effects of fungi and other entomopathogens on reproductive parameters [16,29]. This aligns with a generally good ecotoxicological profile being recognized for *B. bassiana* that is compatible not only with chrysopids, but with a variety of other predators such as coccinellids and hemipterans, and with soil insects such as collembolans [9,30,31].

According to our study, no specific risks resulted from the exposure of *C. lucasina* larvae to conidia of *B. bassiana* strain ATCC 74040, which supports the simultaneous use of these two agents for biological control (predator and fungus) and highlights the safety of this *B. bassiana* strain for wild chrysopid populations. In contrast, there are also studies that report a deleterious effect of *B. bassiana* on different chrysopid species [32,33]. In addition to a variable degree of susceptibility of different host species, different studies have employed diverse strains of the fungus, which can be associated with variable virulence against different targets [34,35,36].

Studies on the compatibility of a *B. bassiana* strain with chrysopids merit further exploration into the combined use of entomopathogens and predators, as demonstrated in studies with the predators *Harmonia axyridis* (Pallas) (Coleoptera: Coccinellidae) and *C. carnea* that were inoculated with *B. bassiana* conidia to disseminate the fungus in the environment [37]. On the other hand, every specific use should be accurately studied, especially in field conditions where the complexity of the relationships among species and the presence of other stress factors may lead to new or unexpected effects [38].

## 5. Conclusions

This investigation contributes to defining the safety and a good ecotoxicological profile of a commercially available *B. bassiana* strain that is largely employed as a valuable biosolution for pest management, according to the principles of eco-sustainability. The evidence for the compatibility of this *B. bassiana* strain with *C. lucasina* arising from this study also provides practical information for the combined employment of these pest management agents in crop systems.

## Figures and Tables

**Table 1:** Means (± SE) of larval and pupal development time, percentage of adult emergence, and sex ratio of two *Chrysoperla lucasina* strains (laboratory and wild) exposed to *Beauveria bassiana* conidia at different concentrations.

*Beauveria bassiana* Concentration (Conidia/mL)	Development Time (Days)		Adult Emergence ^c^ %	Sex Ratio (Male %)
	Larvae ^a^	Pupae ^b^		
*Laboratory strain*				
10^7^	16.3 ± 1.26 a ^d^	11.5 ± 0.93 a	92.5 ± 2.50 ab	73.1 ± 5.33 b
10^6^	16.6 ± 1.62 a	11.4 ± 1.19 a	95.0 ± 2.89 a	55.0 ± 5.49 a
10^5^	16.8 ± 1.42 a	11.1 ± 1.01 a	92.5 ± 2.50 ab	59.4 ± 5.24 a
Control	15.8 ± 1.37 a	11.5 ± 1.03 a	97.5 ± 2.50 a	49.4 ± 12.48 a
*Wild strain*				
10^7^	17.5 ± 1.29 a	11.71 ± 0.99 a	80.0 ± 7.07 b	51.6 ± 10.70 a
10^6^	19.0 ± 1.98 a	11.83 ± 1.02 a	82.5 ± 8.54 b	37.5 ± 11.09 a
10^5^	17.8 ± 1.90 a	12.0 ± 0.89 a	85.0 ± 6.45 b	31.5 ± 12.78 a
Control	17.1 ±1.20 a	11.7 ± 0.91 a	85.0 ± 6.45 b	54.6 ± 6.61 a

^a^ calculated from egg hatching to pupation. ^b^ calculated from pupation to adult emergence. ^c^ calculated on the initial number of larvae. ^d^ Different letters in a column indicate significantly different means (two-way ANOVA on transformed data in the case of percentages, followed by the Tukey test, *p* < 0.05).

**Table 2:** Means (± SE) of eggs/female and egg hatching rate of two *Chrysoperla lucasina* strains (laboratory and wild) exposed to *Beauveria bassiana* conidia at different concentrations.

*Beauveria bassiana* Concentrations (Conidia/mL)	N ^a^	Eggs/Female	Egg Hatching ^b^ %
*Laboratory strain*			
10^7^	11	288.4 ± 46.03 ^c^	55.0 ± 8.66
10^6^	17	355.0 ± 54.94	60.0 ± 4.08
10^5^	16	234.8 ± 63.38	62.0 ± 6.29
Control	18	294.5 ± 64.42	70.0 ± 4.08
*Wild strain*			
10^7^	13	211.9 ± 54.27	72.5 ± 11.09
10^6^	16	182.7 ± 31.75	77.5 ± 7.50
10^5^	16	237.6 ± 14.44	67.5 ± 8.53
Control	14	254.1 ± 57.32	55.0 ± 6.45

^a^ Number of ovipositing females. ^b^ Egg hatching was determined on four occasions during the experiment. Mean values are reported. ^c^ Means in each column are not significantly different (two-way ANOVA on transformed data in the case of percentages, *p* > 0.05).

## Data Availability

Data are contained within the article.
